# Ultrasound-Guided Percutaneous Core Needle Biopsy of Peripheral Pulmonary Nodules ≤ 2 cm: Diagnostic Performance, Safety and Influence Factors

**DOI:** 10.3389/fonc.2021.671884

**Published:** 2021-05-14

**Authors:** Weijun Huang, Jieyi Ye, Yide Qiu, Weiwei Peng, Ninghui Lan, Ting Huang, Yinghui Ou, Xiaoyun Deng, Yingjia Li

**Affiliations:** ^1^ Department of Medical Ultrasonics, Nanfang Hospital, Southern Medical University, Guangzhou, China; ^2^ Division of Interventional Ultrasound, Department of Medical Ultrasonics, Foshan First People’s Hospital (The Affiliated Foshan Hospital of Sun Yat-sen University), Foshan, China

**Keywords:** peripheral pulmonary lesions, core needle biopsy, ultrasound-guided, diagnostic performance, safety

## Abstract

**Purpose:**

To evaluate diagnostic performance and safety of ultrasound-guided needle biopsy in the diagnosis of peripheral pulmonary nodules (PPLs) ≤ 2 cm, and the influence factors of sample adequacy and safety.

**Materials and Methods:**

194 patients (99 men, 95 women; mean age, 56.2 ± 13.7 years) who received biopsy for PPLs ≤ 2 cm between January 2014 to January 2019 were included. Variables including patient demographics, lesion location, lesion size, presence of lesion necrosis, presence of emphysema on CT, patient position, biopsy needle size and number of needle passes were recorded. Univariate analysis and multivariate logistic regression analysis were performed to explore the influence factor of sample adequacy and safety.

**Results:**

Biopsy specimens were adequate for diagnosis in 161/194 (83%) cases; the diagnostic accuracy was 81.4% (158/194). The overall complication rate was 8.8% (17/194), including pneumothorax, hemoptysis and pleural effusion, which occurred in 2.1% (4/194), 5.2% (10/194), and 1.5% (3/194) of patients, respectively. The incidence of pneumothorax in the 16-gauge-needle group were significantly higher than that of the 18-gauge-needle group (5.6% *vs* 0%, *P*=0.018). Adequate sampling of 16-gauge and 18-gauge needles were achieved in 90.3%(65/72) and 78.7%(96/122) cases, respectively. Multivariate logistic regression analysis revealed needle size (16-gauge *vs* 18-gauge) was an independent influence factors of sample adequacy (*P*=0.015, odds ratio=3.419). A receiver operating characteristic curve was plotted and the area under the curve was 0.774.

**Conclusion:**

US-guided percutaneous needle biopsy is a feasible and safe technique for small PPLs ≤ 2 cm. Needle size is an independent influence factor of sample adequacy and post-procedure pneumothorax. Sixteen-gauge needle has the advantage of achieving adequate sample for pathological analysis, though the risk of pneumothorax should be alerted.

## Introduction

With the application of computed tomography (CT) and advent of lung cancer screening in recent years, peripheral pulmonary lesions (PPLs) has been more frequently detected (1). PPLs are defined as lesions directly in contact with the chest wall without an intervening aerated lung (2). PPLs have an echogenic texture and a sharply defined border due to a strong reflective interface between the aerated lung and the lesion on ultrasound (US) (3). Percutaneous core needle biopsy plays an important role in diagnosing PPLs, as a precise diagnostic procedure is necessary for use in determining appropriate management (4). Compared with CT-guided procedures, US-guided percutaneous needle biopsy has some advantages such as free of radiation exposure, real-time monitoring, convenience and avoiding vessels with Color Doppler imaging (5). Therefore, ultrasound has the strength to be a feasible and reliable guiding tool as an alternative to CT.

In China, US-guided biopsy is usually preferred for PPLs, while CT-guided procedures are mainly recommended for lesions that cannot be displayed by ultrasound (6). Although CT-guided procedures have been well established in a previous study with a similar diagnostic rate compared with US-guided procedures for PPLs, a higher incidence of postprocedural pneumothorax were observed (7). Currently, percutaneous US–guided core needle biopsy plays an increasingly important role in the management of PPLs (8). Some published studies had shown the feasibility and efficacy of US-guided percutaneous biopsy for PPLs (2, 5, 6, 9–11). It was reported that US-guided procedures could achieve a high success rate of 84%~96% (2), a pneumothorax rate of 1%~6%, and a hemorrhage rate of 3.3%~5.1% (2, 6, 12).

However, the increased number of small PPLs detected at CT screening has prompted a new challenge in management (8). Although peripheral non-small cell lung cancer (NSCLC) ≤2 cm without nodal and distant metastasis is classified as T1 stage, tumor size >2 cm is considered a significant indicator of visceral pleural invasion (13). As a result, early diagnosis is crucial for improving prognosis. Regular and repeated CT examinations to evaluate the change of volume of the PPLs is one of the remedies for management (14). However, when PPLs were recognized with a size increase follow-up CT examination, a pathologic diagnosis was required. Adequate tissue acquisition for histologic and molecular characterization of PPL is paramount. Nevertheless, small PPLs was reported to be associated with lower diagnostic accuracy in biopsy (5). To the best of our knowledge, data about diagnostic performance and safety of US-guided biopsy for small PPLs are still deficient. Most previous studies included cases with medium or large PPLs and did not systematically provide detailed analysis on small PPLs due to limited sample size, nor the procedures were performed under CT-guidance (2, 5, 6, 9). To find out the influence factors of efficacy and safety would provide critical information to improve the diagnostic yield and lower the complication rate in biopsy for small PPLs. Therefore, the purpose of this study was to evaluate diagnostic performance and safety of ultrasound-guided needle biopsy in the diagnosis of PPLs ≤ 2 cm, and the influence factors of sample adequacy and safety.

## Materials and Methods

### Patients

**Figure 1:**
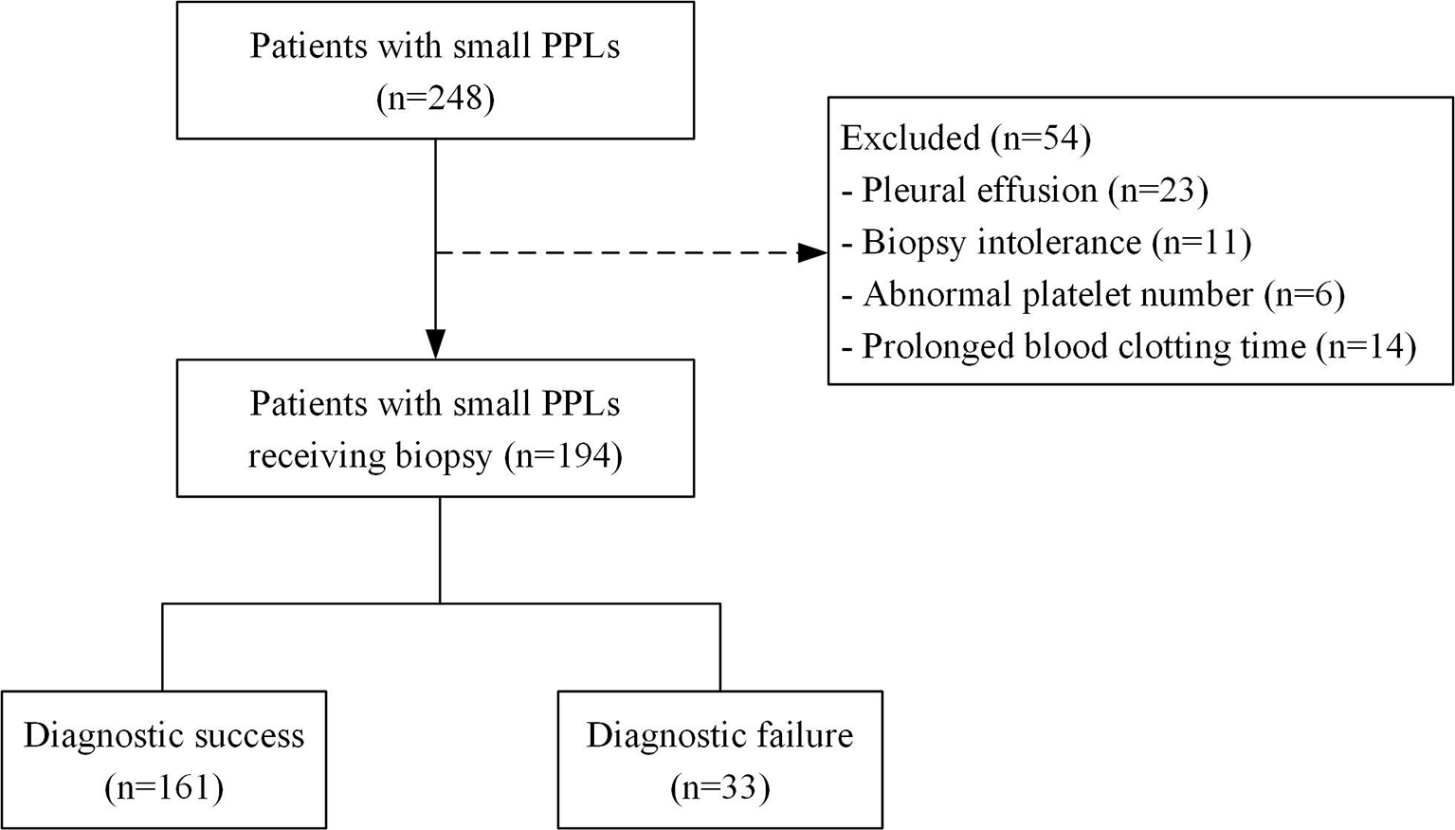
Flow chart of patient inclusion. PPLs, peripheral pulmonary lesions.

From January 2014 and January 2019, a cohort of 194 consecutive patients who received US-guided percutaneous biopsy procedures for PPLs ≤ 2 cm at our hospital were included in the study. Exclusion criteria for biopsy were as follows: patients with pleural effusion, biopsy intolerance resulted from severe cough or cardiopulmonary dysfunction, and abnormal platelet number or prolonged blood clotting time. The Flow chart of patient inclusion was shown in **Figure 1**.

### Data Collection

The institutional review board of our hospital waived the requirement to obtain informed consent and approved the protocol for this retrospective study. Diameters of all nodules were measured as the long-axis measurement on lung window settings in the axial plane on CT. Data review and collection was performed to confirm patient demographics (age, gender), previous cancer history, lesion location, lesion size, presence of lesion necrosis, presence of emphysema on CT, patient position, biopsy needle size, number of needle passes, histopathology reports and post-procedure complications.

### US-Guided Biopsy Techniques and Procedures

All patients received CECT examinations in two weeks before the biopsy procedure. US-guided biopsies were performed using a MyLab Twice machine (Esoate, Genoa, Italy) equipped with a convex array probe CA541 (frequency range from 1 to 8 MHz) by a sonographer with 10 years’ experience in thoracic and interventional ultrasound. Biopsy was performed using needles with a core of 18-gauge or 16-gauge (Bard, Arizona, USA). The protruding needle (22 mm) with biopsy notch (16 mm length) was the same in all cases. Biopsy needle size and biopsy technique were dictated by operator discretion.

**Figure 2:**
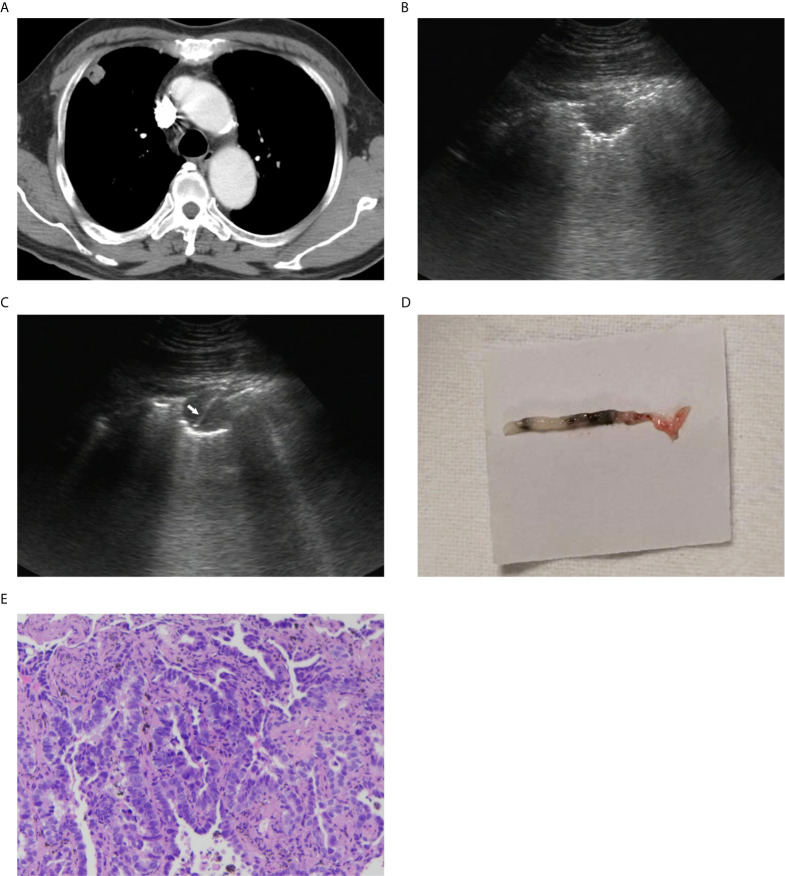
A 67-year-old man with a peripheral lung lesion. Ultrasound-guided percutaneous core needle biopsy was performed. **(A)** Contrast-enhanced CT image showed a lung lesion located in right upper lobe with pleural contact. The diameter of the lesion was 19 mm. **(B)** The lesion manifested as low-echoic on baseline ultrasound. **(C)** The 16-gauge needle track during the biopsy procedure was shown on the image (white arrow). **(D)** Color photograph of the biopsy sample. **(E)** Pathological evaluation showed that heterotypic cells were arranged in tubular or adenoid structures (HE staining; 10×).

According to the location of PPLs, all patients were managed in supine, lateral or prone positions. After administration of local antiseptics and anesthetic, the biopsy needle was inserted with a freehand approach. Real-time color Doppler imaging was used to avoid vessels. The needle could be clearly seen throughout its whole course. Needle insertion angle along linear path was kept as close to 90° as possible. Biopsies were performed during patient’s breath-holding. Samples adequacy were assessed visually, and biopsy was repeated until adequate specimens were achieved. Biopsies were performed 1 to 4 times as tolerated by the patient. The tissue was conserved in formalin and sent for pathological analysis (**Figure 2**).

### Complication Evaluations

After biopsy, patients were monitored for about 30 minutes. Patients were mandated to kept in bed without movement for at least 8 hours. Patients received chest radiograph and ultrasound examination 1 hour after biopsy to detect the presence of complications including pneumothorax and pleural effusion. Further follow-up radiographs were performed when needed (15, 16). A large pneumothorax is identified when the rim between the lung margin and inner chest wall more than 2 cm, which was measured on a chest radiograph at the level of the hilum (17). Hemorrhage could present as hemoptysis and pleural effusion. The severity of pleural effusion was based on the width of free fluid area on ultrasonography, and major pleural effusion was defined as width ≥2 cm. Chest tube insertion was required in cases with large pneumothorax and major pleural effusion, and removed when complication was relieved. Chest radiograph was taken before discharge.

### Pathological Evaluations

Two pathologists with 10 years of experience evaluated the images together and made a final determination. Biopsy results were classified as malignant disease, benign disease, or nondiagnostic. Results were considered nondiagnostic when the tissue was deemed inadequate for diagnosis or showed few atypical cells. A malignant disease was confirmed on when the surgical pathology was the same as the biopsied one. A benign disease was confirmed when the surgical pathology was the same as the biopsied one or when the lesion regressed or remained stable for at least 2 years’ follow-up (18). Nondiagnostic cases were sent for re-biopsy or surgical biopsy.

### Statistical Analysis

Data were reported as percentage or mean ± standard deviation, as appropriate. Differences in quantitative variables were detected with independent sample t-test or Mann–Whitney U-test. Differences in categorical variables were detected using χ^2^ -test or Fisher’s exact test. *P*<0.05 was considered to indicate a statistically significant difference.

A multivariate logistic regression model was built to identify independent influence factors associated with biopsy sample adequacy. An Enter model was used and all variables were sent for binary logistic regression analysis. As lesion location and patient position are unordered categorical variables, dummy variables were used in multivariate logistic regression analysis. For patient position, supine was used as reference to set up dummy variables (X1, lateral=1; X2, prone=1). For lesion location, left upper lobe was used as reference to set up dummy variables (X1, left lower lobe=1; X2, right upper lobe=1; X3, right middle lobe=1; X4, right lower lobe=1). A receiver operating characteristic (ROC) curve was plotted, and the area under the ROC curve was calculated to determine the predictive value of the logistic regression model. Statistical analyses were performed using the SPSS 16.0 software package (IBM, NY, USA).

## Results

### Patients and Lesions Profile

A total of 194 patients were included in the current study. There were 99 (51.0%) men and 95 (49.0%) women, with a mean age of 56.2 ± 13.7 years (range 18~80 years). The average diameter of the PPLs was 1.6 ± 0.4 cm (ranging from 0.6 cm to 2.0 cm). The detailed baseline characteristics and lesions profile were displayed in **Table 1**.

**Table 1:** Patients characteristics and lesion profiles (n = 194).

Characteristics	n = 194
Age (years)	
Mean ± SD (range)	56.2 ± 13.7 (18~80)
Gender	
Male/female	99/95
Lesion size (cm)	
Mean ± SD (range)	1.6 ± 0.4 (0.6~2.0)
Lesion location	
Left upper lobe	31
Left lower lobe	49
Right upper lobe	47
Right middle lobe	14
Right lower lobe	53
Presence of necrosis	
Yes/No	40/154
Patient position (%)	
Supine	81
Prone	29
Lateral	84
Emphysema on CT	
Yes/No	17/177
Needle size (gauge)	
16/18	72/122
Needle passes	
1 time	28
2 times	117
≥3 times	49

### Diagnostic Performance of Biopsy and Influence Factors

The rate of adequate sample for pathological analysis was 83.0% (161/194). Among all biopsies, 74 lesions were identified as malignant disease: 48 adenocarcinoma, 12 metastatic cancer, 7 squamous cell carcinoma, 3 small cell lung cancer, 3 sarcoma and 1 poorly-differentiated carcinoma. On the other hand, 87 lesions were categorized as benign disease: 36 tuberculosis, 24 chronic inflammation, 9 pneumonia, 7 organizing pneumonia, 5 granulomatous inflammation, 4 cryptococcosis, 1 aspergillosis and 1 pneumoconiosis. The remaining 33 biopsy samples were categorized as non-diagnostic. **Table 2** provides the pathologic diagnoses for the biopsies.

**Table 2:** Pathological results of ultrasound-guided biopsy for 194 PPLs.

Biopsy results	No. (%) of patients
Malignant disease	74 (38.2)
Squamous cell carcinoma	7 (3.6)
Adenocarcinoma	48 (24.7)
Small cell carcinoma	3 (1.6)
Metastasis	12 (6.2)
Sarcoma	3 (1.6)
Poorly-differentiated carcinoma	1 (0.5)
Benign disease	87 (44.8)
Tuberculosis	36 (18.6)
Pneumonia	9 (4.6)
Chronic inflammation	24 (12.4)
Organizing pneumonia	7 (3.6)
Pneumoconiosis	1 (0.5)
Aspergillosis	1 (0.5)
Cryptococcosis	4 (2.0)
Granulomatous inflammation	5 (2.6)
Nondiagnostic	33 (17.0)

PPLs, peripheral pulmonary lesions.

Of the 74 lesions diagnosed as malignant, 62 (83.8%) were confirmed by surgical resection. Twelve (16.2%) were confirmed based on the results of biopsy for PPLs and the biopsies for other sites, and both presented as the same histological results. Of the 87 lesions diagnosed as benign, 2 lesions (2.3%) were confirmed through surgical resection. Overall, 34 lesions (39.1%) were confirmed as benign based on biopsy results and regression or disappearance of the lesion at follow-up. Fifty-one lesions (58.6%) were confirmed as benign owing to absence of change in size for more than 2 years.

Seventy-four malignant lesions and 84 of the 87 benign lesions were diagnosed correctly. Three cases were diagnosed as benign based on biopsy result but were confirmed as malignant after a surgical biopsy or a bronchoscopy biopsy. The overall diagnostic accuracy was 81.4% (158/194). Of the 33 lesions diagnosed as non-diagnostic, 12 (36.4%) were confirmed by surgical resection, 18 (54.5%) were confirmed by surgical biopsy and the remaining 3 (9.1%) were confirmed by bronchoscopy biopsy.

Univariate analysis showed that the rate of sample adequacy was significantly higher in 16-gauge-needle group than that of the 18-gauge-needle group (90.3% *vs* 78.7%, *P*=0.038). There was no significant difference in age, gender, lesion location, lesion size, presence of lesion necrosis, patient position, presence of emphysema on CT and times of needle passes between sample-adequate group and sample-inadequate group (all *P*>0.05) (**Table 3**).

**Table 3:** Univariate analysis of influence factors for sample adequacy of ultrasound-guided percutaneous core needle biopsy for PPLs ≤ 2 cm.

Variables	Sample adequacy for pathological diagnosis		*P* value
	Confirmed cases (n = 161)	Unconfirmed cases (n = 33)	
Age (years)	56.5 ± 13.9	54.7 ± 12.7	0.491
Gender			
Male/female	80/81	19/14	0.409
Lesion size (cm)	1.6 ± 0.4	1.5 ± 0.4	0.708
Lesion location			
Left upper lobe	27	4	0.086
Left lower lobe	35	14	
Right upper lobe	40	7	
Right middle lobe	14	0	
Right lower lobe	45	8	
Presence of necrosis			
Yes/No	36/125	4/29	0.185
Patient position (%)			
Supine/Lateral/ Prone	71/21/69	10/8/15	0.166
Emphysema on CT			
Yes/No	16/145	1/32	0.347
Needle size (gauge)			
16/18	65/96	7/26	0.038
Needle passes			
1 time	23	5	0.842
2 times	96	21	
≥3 times	42	7	

PPLs, peripheral pulmonary lesions.

**Figure 3:**
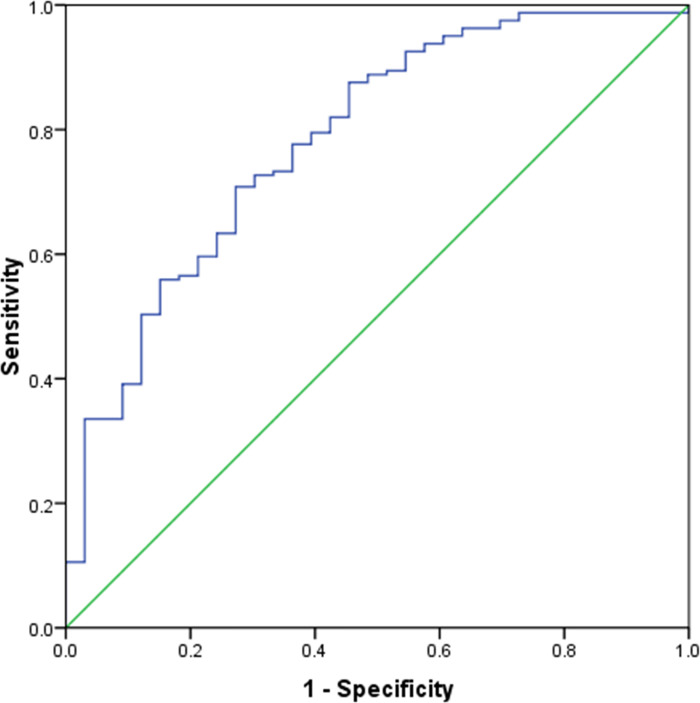
Receiver operating characteristic curve analysis of predictive value of the multivariate logistic regression model. The area under the curve was 0.774.

All variables were sent for binary multivariate logistic regression analysis. Multivariate logistic regression analysis revealed that only needle size (16-gauge *vs* 18-gauge) (*P*=0.015, OR = 3.419, 95% CI = 1.264-9.249) was an independent influence factor of sample adequacy. Details of multivariate logistic regression analysis results are summarized in **Table 4**. An ROC curve (**Figure 3**) was plotted to evaluate the predictive value of the logistic regression model. The area under the ROC curve was 0.774 (*P*<0.001, 95% CI = 0.686–0.862).

**Table 4:** Multivariate logistic regression analysis of influence factors for sample adequacy of ultrasound-guided percutaneous core needle biopsy for PPLs ≤ 2 cm.

Variables	B	OR	95%CI	*P* value
Age (y)	0.004	1.004	0.972-1.036	0.828
Gender (female *vs* male)	0.366	1.442	0.621-3.351	0.395
Lesion location	NA	NA	NA	0.323
X1 (left lower lobe=1)	-1.174	0.309	0.080-1.203	0.090
X2 (right upper lobe=1)	-0.670	0.512	0.112-2.343	0.388
X3 (right middle lobe=1)	19.698	3.59×10^8^	NA	0.998
X4 (right lower)	-0.158	0.853	0.210-3.460	0.824
Lesion size (cm)	0.023	1.023	0.909-1.151	0.708
Emphysema by CT (no *vs* yes)	-1.809	0.164	0.018-1.497	0.109
Presence of necrosis (no *vs* yes)	-0.896	0.408	0.124-1.340	0.139
Patient position	NA	NA	NA	0.129
X1 (lateral=1)	-1.336	0.263	0.071-1.274	0.065
X2 (prone=1)	-0.440	0.644	0.236-1.756	0.390
Needle size (16-gauge *vs* 18-gauge)	1.229	3.419	1.264-9.249	0.015*
Needle passes (times)	0.368	1.445	0.771-2.705	0.250

PPLs, peripheral pulmonary lesions; OR, odds ratio; CI, confidence interval; NA, not available. *P < 0.05.

### Complications of Biopsy and Influence Factors

The overall post-procedure complication rate was 8.8% (17/194). None of these incidents resulted in permanent severe sequelae or death. The overall rate of hemorrhage was 6.7% (13/194), including 10 cases of hemoptysis and 3 cases of pleural effusion. No major hemorrhage occurred. All cases were self-limited and recovered after conservative treatment including rest, placement in a puncture-site-down position and use of Tranexamic acid. No significant difference was detected in respect to age, gender, lesion location, presence of lesion necrosis, patient position, presence of emphysema on CT, needle size and times of needle passes between complication group and non-complication group, hemoptysis group and non-hemoptysis group, pleural-effusion group and non-pleural-effusion group (all *P*>0.05).

Totally, pneumothorax occurred in 4 out of 194 patients (2.1%), and all cases were recorded as minor pneumothorax. The patients were monitored in a puncture-site-down position and received nasal oxygen. The rate of pneumothorax in the 16-gauge-needle group were significantly higher than that of the 18-gauge-needle group (5.6% *vs* 0%, *P*=0.018), while no significant difference was detected in respect to age, gender, lesion location, presence of lesion necrosis, patient position, presence of emphysema on CT and times of needle passes between pneumothorax group and non-pneumothorax group (all *P*>0.05) (**Table 5**).

**Table 5:** Univariate analysis of influence factors for safety of ultrasound-guided percutaneous core needle biopsy for peripheral pulmonary lesions ≤ 2 cm.

Characteristics	Total No. (%)	Complication		Pneumothorax		Hemoptysis		Pleural effusion	
	194 (100)	n (%)	*P*	n (%)	*P*	n (%)	*P* value	n (%)	*P*
Age (years)									
≤65	52 (26.8)	5 (9.6)	0.799	2 (3.8)	0.292	3 (5.8)	1.000	2 (3.8)	1.000
>65	142 (73.2)	12 (8.5)		2 (1.4)		7 (4.9)		1 (0.7)	
Gender									
Male	99 (51.0)	5 (5.1)	0.062	1 (1.0)	0.361	2 (2.0)	0.091	1 (1.0)	0.615
Female	95 (49.0)	12 (12.6)		3 (3.2)		8 (8.4)		2 (2.1)	
Lesion location									
Left upper lobe	31 (15.9)	2 (6.5)	0.843	1 (3.2)	0.972	0 (0)	0.242	1 (3.2)	0.402
Left lower lobe	49 (25.3)	3 (6.1)		1 (2.0)		2 (4.0)		0 (0)	
Right upper lobe	47 (24.2)	5 (10.6)		1 (2.1)		4 (8.5)		0 (0)	
Right middle lobe	14 (7.2)	2 (14.3)		0 (0)		2 (14.3)		0 (0)	
Right lower lobe	53 (27.4)	5 (9.4)		1 (1.9)		2 (3.8)		2 (3.8)	
Presence of necrosis									
Yes	30 (15.5)	2 (6.7)	0.528	2 (6.7)	0.189	0 (0)	0.210	0 (0)	1.000
No	164 (84.5)	15 (9.1)		2 (1.2)		10 (6.1)		3 (1.8)	
Patient position (%)									
Supine	81 (41.8)	5 (6.2)	0.191	0 (0)	0.069	5 (6.2)	0.213	0 (0)	0.310
Lateral	29 (14.9)	5 (17.2)		0 (0)		3 (10.3)		1 (3.4)	
Prone	84 (43.3)	7 (8.3)		4 (4.8)		2 (2.4)		2 (2.4)	
Emphysema by CT									
Yes	17 (8.8)	0 (0)	0.374	0 (0)	1.000	0 (0)	0.666	0 (0)	1.000
No	177 (91.2)	17 (9.6)		4 (2.3)		10 (5.6)		3 (1.7)	
Needle size (gauge)									
16	72 (37.1)	9 (12.5)	0.157	4 (5.6)	0.018*	3 (4.2)	0.887	1 (1.4)	0.286
18	122 (62.9)	8 (6.6)		0 (0)		7 (5.7)		2 (1.6)	
Needle passes									
1 time	28 (14.4)	3 (10.8)	0.492	0 (0)	0.438	1 (3.6)	0.539	1 (3.6)	0.462
2 times	117 (60.3)	8 (6.8)		2 (1.7)		5 (4.3)		2 (1.7)	
≥3 times	49 (25.3)	6 (12.2)		2 (4.1)		4 (8.2)		0 (0)	

*P < 0.05.

## Discussion

The current study demonstrated that US-guided percutaneous needle biopsy for small PPLs is feasible and safe, and needle size is the independent influence factor of sample adequacy and post-procedure pneumothorax. In other words, sixteen-gauge needle has the advantage of achieving adequate sample for pathological analysis compared with 18-gauge needle, though the increasing risk of pneumothorax should be alerted.

How to manage patients with PPLs that are 2 cm or smaller is the ultimate question for radiologists and physicians. When physicians consider adopting US-guided percutaneous core needle biopsy for small PPLs, there is no doubt that they will consider the lesion size inevitably lower the diagnostic performance, which has been proved by researchers (5, 11). Some scholars attributed the lower diagnostic performance in small lesions to missed sampling and insufficient sampling (5). Therefore, to acquire adequate tissue sample for pathological diagnosis plays a crucial role in improving diagnostic performance. As we know, repeatability and a larger number of samples may increase the success rate of biopsy. In our study, the only influence factor that affected the sample adequacy of US-guided percutaneous biopsy for small PPLs in univariate and multivariate analyses was the needle size. In our series, 16-gauge needle was applied in 37.1% of cases, yielding a larger tissue sample and improving the success rate of pathological evaluation to about 3.4 folds compared with using an 18-gauge needle. Certainly, cutting groove volume of 16-gauge needle is naturally greater than that of 18-gauge needle. Besides, when minor pneumothorax occurs during biopsy, the gas might effuse to the pleural cavity from the needle tract. As a result, the PPL would be obscured by the gas and affect further sampling. In this situation, a large needle will take advantage over a smaller one in acquiring adequate tissue sample. Many studies have demonstrated that US-guided percutaneous biopsy for PPLs is effective and the accuracy has ranged from 82%~96% in confirmative pathological diagnoses (7, 19–24). In terms of diagnostic accuracy, the overall rate was 81.4% in this study, which was comparable to those of previous studies.

The other consideration is the safety of this invasion biopsy procedure. The common complications are pneumothorax and hemorrhage (including hemoptysis or pleural effusion). In a study of CT-guided needle biopsy for lung lesions, the rates of pneumothorax and bleeding were 17-fold and 6-fold higher in pulmonary lesions ≤ 2 cm (25). On the other hand, US-guided biopsies for PPLs≤ 2 cm were safe with a pneumothorax rate of 2.1% and a hemorrhage rate of 6.7% in the present study. There were no mortalities in all procedures. The results were comparable to previous studies that included various lesion size and reported rates of pneumothorax and hemorrhage ranging from 1%~6% and 3.3%~5.1%, respectively (2, 6, 12). Influence factors regarding patient characteristics, tumor profiles, needle size and needle passage were analysed. In our series, the incidence of pneumothorax was higher using 16-gauge needle (5.6%) rather than 18-gauge needle (0%). For small PPLs, lesion-pleura contact arc length that proposed by Jeon et al. (26) is short. Consequently, when the needles penetrate into visceral pleura, the possibilities of injuring gas-containing pulmonary tissue increase, especially if larger size needles are employed in the biopsy. Larger needle size tends to injure a larger part of lung parenchyma and lead to expanding in the amount of air leakage. As a result, gas escapes from the damaged pleura to form a pneumothorax. Similarly, Geraghty et al. reported needle size had a significant effect on pneumothorax rate in CT-guided biopsy (27). However, the pneumothorax induced by 16-gauge needle in our study were all recognized as minor, and all were discharged after conservation therapy.

Hemorrhage is another concerned issue. Hemoptysis and hemothorax presenting as pleural effusion are two important urgent complications that will interrupt air exchanges in the lungs (28, 29). Except for lesion-related, patient-related and operator-related-factors, procedure-related factors should be paid more attention. Procedure-related factors including needle size, needle pass times and needle insertion angle are well within operator control. In our series, choosing the shortest distances to the PPLs and penetrate the pleural as vertically as possible by a single experienced operator resulted in 5.2% hemoptysis and 1.5% pleural effusion among all cases. The occurrence of hemoptysis and pleural effusion with the use of 16-gauge needle was approximate to that using 18-gauge needle (4.2% *vs* 5.7% and 1.4% *vs* 1.6%, respectively). At present, 18-gauge needles are typically used. For patients, 16-gauge needle could increase the tissue yield without increasing the risk of bleeding compared with 18-guage needle, which was very praiseworthy and valuable. This reason might be that the blood supply of small PPLs is not so abundant, and the risk of penetrating large blood vessels and causing severe hemorrhage is low. In respect to needle passes, no significant correlation between number of needle passes and hemorrhage rate was found in a meta-analysis of influence factor of CT-guided core needle biopsy for lung lesions (30).

Several limitations of this study should be taken into account. First, it is a retrospective study and selection bias was inevitable. Second, the lack of surgical pathology in some cases is an important limitation. In this respect, we used reference standards accepted in clinical practice and routinely used for management decisions. Third, the study was performed in a single center, and some unavailable potential influence factors could not be evaluated, such as relationship between lesions and respiration, the depth of penetration, pulmonary function and smoking history of the patients. Therefore, further validation of results of this study is still warranted.

## Conclusions

In conclusion, US-guided percutaneous needle biopsy for small PPLs is a feasible and safe technique. Needle size is the independent influence factor of sample adequacy and post-procedure pneumothorax. Therefore, 16-gauge needle has the advantage of achieving adequate sample for pathological analysis compared with 18-gauge needle, though the risk of pneumothorax should be alerted.

## Data Availability Statement

The raw data supporting the conclusions of this article will be made available by the authors. Further inquiries can be directed to the corresponding author.

## Ethics Statement

The studies involving human participants were reviewed and approved by Institutional Review Board of First People’s Hospital of Foshan. The patients/participants provided their written informed consent to participate in this study.

## Author Contributions

YDQ, WWP, NHL, TH, YHO, and XYD participated in literature search, data acquisition, data analysis, or data interpretation. WJH, JYY, and YJL conceived and designed the study, and critically revised the manuscript, performed the research, wrote the first draft, collected and analyzed the data. WJH and JYY participated in paper writing and revised the manuscript. All authors contributed to the article and approved the submitted version.

## Funding

This work was supported by the Foshan Engineering Technology Research Center Project [Grant No. 2020001004115].

## Conflict of Interest

The authors declare that the research was conducted in the absence of any commercial or financial relationships that could be construed as a potential conflict of interest.
